# A review of abnormalities in the perception of visual illusions in schizophrenia

**DOI:** 10.3758/s13423-016-1168-5

**Published:** 2016-10-11

**Authors:** Daniel J. King, Joanne Hodgekins, Philippe A. Chouinard, Virginie-Anne Chouinard, Irene Sperandio

**Affiliations:** 1 School of Psychology, University of Birmingham, Edgbaston, Birmingham, West Midlands B15 2TT United Kingdom; 2 Department of Clinical Psychology, Norwich Medical School, University of East Anglia, Norwich Research Park, Norwich, NR4 7TJ United Kingdom; 3 Department of Psychology and Counselling, School of Psychology and Public Health, La Trobe University, Melbourne, Victoria Australia; 4 Psychotic Disorders Division, McLean Hospital, Belmont, MA USA; 5 Harvard Medical School, Department of Psychiatry, Boston, MA USA; 6 School of Psychology, University of East Anglia, Norwich Research Park, Norwich, NR4 7TJ United Kingdom

**Keywords:** Schizophrenia, Visual illusions, Low-level vision, High-level vision

## Abstract

Specific abnormalities of vision in schizophrenia have been observed to affect high-level and some low-level integration mechanisms, suggesting that people with schizophrenia may experience anomalies across different stages in the visual system affecting either early or late processing or both. Here, we review the research into visual illusion perception in schizophrenia and the issues which previous research has faced. One general finding that emerged from the literature is that those with schizophrenia are mostly immune to the effects of high-level illusory displays, but this effect is not consistent across all low-level illusions. The present review suggests that this resistance is due to the weakening of top–down perceptual mechanisms and may be relevant to the understanding of symptoms of visual distortion rather than hallucinations as previously thought.

## Introduction

Visual illusions are a key methodology in vision research to help us understand and make inferences about the mechanisms for creating subjective experiences of the visual world. They place constraints on the processing of a stimulus and allow for the reliable manipulation and quantification of the visual mechanisms the illusion engages (Silverstein & Keane, 2011b). Examining visual illusions in individuals with schizophrenia may provide further insight into how these individuals perceive the world and how their visual perception differs from unaffected individuals. Such insights may further our understanding of the mechanisms underlying psychotic symptoms, such as hallucinations and visual distortions. This article investigates whether or not individuals with schizophrenia have a different susceptibility to visual illusions compared to unaffected individuals, and what this may tell us about the disorder.

### Perceptual organisation in schizophrenia

Research has typically shown that patients with schizophrenia exhibit specific abnormalities in lower (Brenner, Wilt, Lysaker, Koyfman, & O’Donnell, 2003; Chen et al., 1999; Green, Nuechterlein, & Mintz, 1994; Kantrowitz, Butler, Schecter, Silipo, & Javitt, 2009) as well as higher level visual processing, such as facial emotion recognition (Edwards, Jackson, & Pattison, 2002; Schneider et al., 2006). Whilst auditory hallucinations are prevalent in patients with psychotic disorders, visual hallucinations occur relatively frequently, with a recent study showing a point-prevalence (the proportion of the given population experiencing the symptom over a fixed temporal window) of 27 % for those with schizophrenia (Waters et al., 2014). Although less well studied, symptoms of visual distortions are also present in schizophrenia, and defined as perceived distortions of real external stimuli (Chouinard & Miller, 1999). Visual distortion symptoms contrast with hallucinations, which are not based on real external stimuli. It must be noted that these types of distortion referred to here are separate from the effects of the methodology of illusory displays that this article reviews; however, they may still inform one another. Anomalies of perception are also an important feature for at-risk mental states and prodromal psychosis (Yung & McGorry, 1996). When taken together, these visual processing and perceptual abnormalities justify further decomposition of visual perceptual abilities, which should be investigated as a functional abnormality in schizophrenia.

Perceptual organisation is one of several perceptual abilities affected in schizophrenia (Butler, Silverstein, & Dakin, 2008; Place & Gilmore, 1980; Silverstein & Keane, 2011a; Uhlhaas & Silverstein, 2005; Uhlhaas, Phillips, Mitchell, & Silverstein, 2006a). Perceptual organisation involves processes vital to assigning salience to individual features of the visual input and binding them to create a coherent, unified percept (der Helm, 2014; Silverstein & Keane, 2011a).

Perceptual organisation processes can be grossly categorised into low-level and high-level integration, depending on what structures in the brain are thought to be relatively more engaged. For the purpose of this review, we refer to low-level processes as those that occur at the level of V1 and earlier, whereas higher processes are those that occur later than V1. This is not to say that either forms of integration occur only at these early or late structures of the sensory system, with bottom–up or top–down influences acting upon both processes, but that the bulk of the processing required occurs there.

In terms of vision, low-level integration refers to the combination of visual signals mediated by the early structures of the visual system. Although this integration could still be modulated by top–down mechanisms, it is less dependent on cognitive control than high-level integration processes. Low-level integration could occur as early as at the level of the retina. The centre-surround properties of a retinal ganglion cell responding antagonistically to light in the centre and the surround of its receptive field (or vice versa) represents an example of low-level integration. Another example would be the further integration of this information at the level of the primary visual cortex, whose cells have centre-surround properties for bars of light (Hubel & Wiesel, 1962).

Conversely, high-level integration is more dependent on cognitive control mechanisms mediated by higher-order brain structures as evidenced from the influences of prior knowledge and culture on visual perception. For example, it has been demonstrated that some visual illusions, such as the Ebbinghaus illusion (see Table 1), develop with age (Doherty, Campbell, Tsuji, & Phillips, 2010), are weaker in African remote cultures (de Fockert, Davidoff, Fagot, Parron, & Goldstein, 2007) and enhanced in East Asian populations (Caparos et al., 2012). It is unlikely that the processing of conceptual knowledge about the world is primarily driven by the retina, the lateral geniculate nucleus, or the primary visual cortex, albeit they could still be modulated by top–down influences (Sperandio & Chouinard, 2015).

**Table 1:**
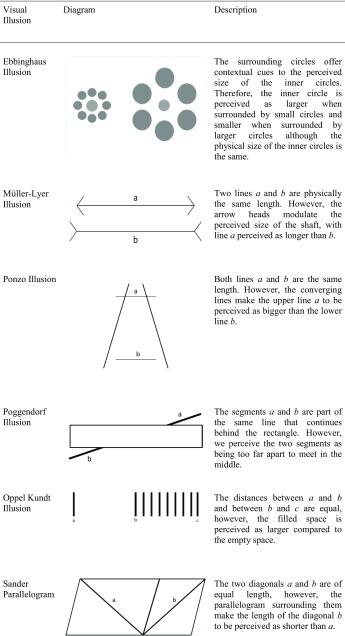

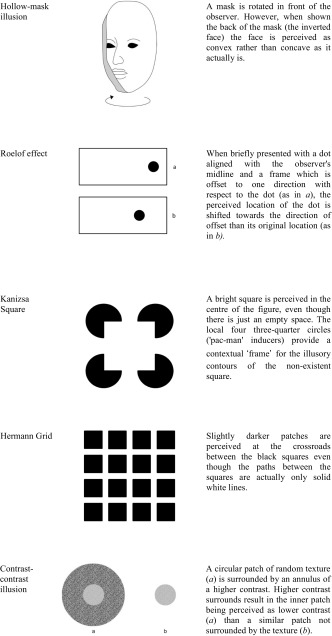

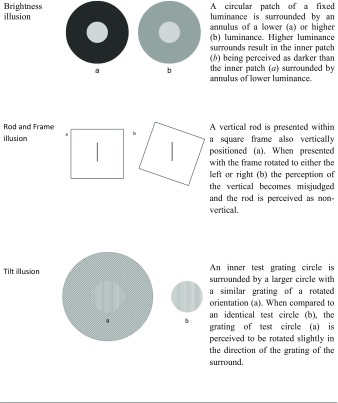
Diagrams and descriptions of visual illusions described in this article

In a review of studies from 2005 to 2010, converging evidence from behavioural, fMRI and EEG studies was identified in support of a deficit in visual perceptual organisation in schizophrenia (Silverstein & Keane, 2011a). Of the 27 studies selected for review, 25 indicated a deficit in perceptual organisation at some level. They also highlighted that some evidence suggests that the abnormalities in perceptual organisation are driven by the deficits that are apparent in bottom–up processing. Interestingly, this same review selected to include data from the Ebbinghaus illusion, alongside a number of other behavioural tasks to assess perceptual organisation processes. This illusion was chosen as evidence of how prior knowledge can affect perceptual organisation or, in the case of schizophrenia, does not affect this mechanism as much. As outlined above, a number of illusions operate by utilising adaptive processes which govern perceptual organisation.

The use of visual illusion methodologies to understand perceptual organisation is informative. This is especially true given that different visual illusions most certainly engage different neural and cognitive operations (Chouinard, Noulty, Sperandio, & Landry, 2013; Chouinard, Unwin, Landry, & Sperandio, 2016; Gregory, 1997), although these mechanisms remain to be more precisely identified. It then follows that the inclusion of multiple visual illusions in a review of perceptual organisation in schizophrenia can lead us to a more detailed qualitative analysis about the nature of some of the abnormalities pertaining to perceptual organisation in schizophrenia.

This review aims to provide a qualitative review of the literature regarding visual illusion susceptibility in populations with schizophrenia. Relevant articles for this review were identified using the PubMed database, with “schizophrenia AND visual illusions” and “schizophrenia AND illusions” as keywords. The current review outlines findings that suggest susceptibility to some but not all low-level integration illusions whilst a more consistent resistance to high-level illusions was found. This suggests that previous bottom–up accounts of abnormal perceptual organisation in schizophrenia do not parsimoniously explain abnormalities in visual perception of these illusions; there seems to be a much more pronounced deficit to higher-level processes.

### Visual illusions as a methodology

Although visual illusions alter our perception of physical reality, they demonstrate how the brain uses specific mechanisms that are highly adaptive, such as those that allow for perceptual constancies (Gregory, 1963). For instance, we need size constancy to have a relatively stable experience of the perceived size of an object whose image on the retina changes continuously with variations in distance. The Ponzo illusion is an illusion that is widely believed to rely on these mechanisms (further discussion will be provided below). These adaptive mechanisms which govern our visual experience ultimately form part of our perceptual organisation, providing rules by which to structure the visual input we receive into an understandable pattern.

Visual illusions can also provide us with insights into how these mechanisms may or may not be affected in a disorder with perceptual anomalies, such as schizophrenia. This is because, in the typical population, they produce a reliable and predictable dissociation between the retinal image of an object and its perception, that can be compared with the disorder (Dima et al., 2009). This review will focus on pictorial illusions. The number of papers published in this field alone has surged in recent years and deserves its own review. We will not cover other types of illusions, such as multisensory illusions and haptic illusions which are covered elsewhere (Ciszewski, Wichowicz, & Krzysztof, 2015; Notredame, Pins, Deneve, & Jardri, 2014), and are likely to rely on differing cognitive apparatus than that of other pictorial illusions (Sperandio & Chouinard, 2015). Table 1 provides short descriptions and illustrations of the types of illusions mainly covered in this article.

Contrast illusions form one class of low-level integration illusions (see Table 1). This class of illusions can be explained by means of lateral inhibition of centre-surround antagonistic cells that operate at the level of the retina, the thalamus or early cortical structures. These structures are better placed than higher-order structures to perform *gain control*, a mechanism that optimally distinguishes between two regions in the visual scene with different contrasts at the point where the two meet so that they are perceived as more different than they actually are (Spillmann, 1994) .

In contrast, other illusions are more cognitively demanding and rely on the processing of more complex features of a visual stimulus, as opposed to the standard features encoded at the level of the retina or lateral geniculate nucleus. For instance, the Ponzo illusion requires the brain to interpret that the linear depth cues depict changes in apparent distance in the visual scene. When this illusion is displayed monocularly, its magnitude is equivalent to when displayed dichoptically (where the contextual lines and the object are each displayed to separate eyes; Song, Schwarzkopf, & Rees, 2011), suggesting that the illusion is mainly processed by brain regions that contain binocular neurons (i.e. from V1 onwards). Thus, this illusion is more reliant on higher-level cortical processes (Song et al., 2011) and can therefore be considered as a type of high-level integration illusion.

Whilst low-level and high-level integration are not truly dichotomous and independent of one another, comparing and contrasting perceptual phenomena that are thought to be mediated more by one than the other could provide insight into how the brain in schizophrenia organises visual perceptual signals in such a way that leads to anomalous perceptual experiences, particularly visual distortion symptoms and visual hallucinations. Thus, it is important for the literature in this area to consider both of these perceptual processes in order to infer at which stage of visual information processing these abnormalities might occur.

For example, Dyde and Milner (2002) compared ‘posting’-based movements (i.e. reaching a slotted target with a card held in the hand as if posting a letter in a mailbox) in the context of tilt as well as the rod and frame illusions (see Table 1). In the tilt condition, participants had to match the angle of tilt when making their posting movement. Whilst the simultaneous tilt illusion is thought to be a low-level illusion originating in V1, an early cortical area, the rod and frame illusion is a context-based illusion that is assumed to be computed much later in the processing of visual information. When comparing the effects of the two illusions, it was found that the simultaneous tilt illusion had no effect on the posting action, whereas the rod and frame illusion reduced the accuracy of the posting movements, suggesting that the posting action originates in the dorsal stream of visual processing and can be selectively affected by illusions that take place at higher-levels in the visual system.

This example is a powerful demonstration of how comparing low- and high-level illusions with each other can inform us not only about which stages of visual processing are involved in separate tasks but also the different types of mechanisms which may be deficient. Such a paradigm could be potentially relevant in visual studies performed in schizophrenia. By identifying the stage of processing which is maladaptive, we can have a better understanding of the functional outcomes and symptomology associated with the disorder.

## Visual illusions and schizophrenia

The majority of studies investigating the effect of visual illusions in patients with schizophrenia have found a reduced susceptibility compared to healthy controls (see Table 2). As we will now discuss, this provides some key insights for understanding atypical visual perception in schizophrenia.

**Table 2:** Results for specific types of illusions in schizophrenia

Illusion	Reference	Sample	PANSS	BPRS	Response type	Results
Ebbinghaus illusion	Uhlhaas, Phillips, Schenkel, & Silverstein, 2006b	40 SCH (6 F, 34 M, *M* = 38.4 years, 12 disorganised SCH, 36 non-sisorganised) 26 Non-psychotic psychiatric controls (10 F, 16 M, *M* = 36.7 years)	Disorganised = 15.1, 19.2, 91.5 Non-disorganised = 11.4, 16.2, 71.0	N/A	Forced choice	Disorganised SZ group were significantly less susceptible to the illusion than non-disorganised and control groups.
	Horton & Silverstein, 2011	65 Chronic SCH patients 34 Deaf (26 SCH, 8 SZ-A, High in affective symptoms) 31 Hearing (24 SCH, SZ-A, high in thought disorder symptoms)	N/A	Illness severity Deaf = .64 Hearing = .65	Forced choice	Magnitude of illusion is significantly less in those participants that had disorganised symptoms compared to those that did not Magnitude less in hearing patients compared to deaf patients due to experience-based neuro-plastic changes
	Yang et al. 2013a	30 SCH (37 % F, *M* = 41 years) 23 HC (48 % F, *M* = 39 years)	N/A	13	Forced choice	No difference between SCH and HC
	Tibber et al., 2013	24 SCH (8 F, 16 M, *M* = 40.0) 24 HC (8 F, 16 M, *M* = 38.2)	14.4, 17.0, 60.9	N/A	Forced choice	Patients with schizophrenia, had reduced contextual biases for size, meaning they were less susceptible to the illusion than HC.
Muller-Lyer illusion	Kantrowitz et al., 2009	38 Chronic SCH/SCH-A (*M* = 37.3 years) 28 HC (*M* = 36.5 years)	N/A	40.9	Forced choice	Magnitude of the illusion greater in patients with SZ, compared to controls
	Tam, Sewell, & Deng, 1998	26 SCH (13 F, 13 M; 9 predominantly positive symptoms; 8 predominantly negative symptoms; 9 with no predominance of positive or negative symptoms) 10 HC (5 F, 5 M)	N/A	N/A	Size-judgment task	Patients were more susceptible to the Muller-Lyer than controls (no reported test of significance)
	Weckowicz & Witney, 1960	27 chronic SCH (33 % hebephrenic, 22 % paranoid, 30 % undifferentiated, 11 F, 16 M, 25–55 years) 28 HC (11 F, 17 M, 19–71 years)	N/A	N/A	Forced choice	Magnitude of the illusion greater in patients with schizophrenia compared to controls
	Diržius, Liutkevičius, Žukauskait, Leskauskas, & Bulatov, 2013	4 Paranoid SCH in remission (1 F, 3 M, 21–44 years) 5 HC (1 F, 4 M, 22–56 years)	N/A	N/A	Size-judgment task	Magnitude was significantly greater in patients with schizophrenia for 5 of 40 different inner-angles for the wings of the illusionary-stimulus.
	Capozzoli & Marsh, 1994	15 Chronic, paranoid SCH Inpatients 14 HC	N/A	N/A	Forced choice	Magnitude of the illusion greater in patients with schizophrenia compared to controls
	Letourneau, 1974	5 Paranoid SCH (*M* = 47.6 years) 5 Simple SCH (*M* = 46.3 years)	N/A	N/A	Size-judgment task	No significant differences between paranoid SZ patients and patients with simple SZ
	Parnas et al., 2001	10 Chronic SCH (3 F, 7 M, *M* = 28.7 years) 9 First-admission SCH (5 F, 4 M, *M* = 25.1 years) 10 Prodromal SCH (7 F, 3 M, *M* = 27.5 years) 14 HC (8 F, 6 M, *M* = 31.9 years)	N/A	N/A	Forced choice	Patients with prodromal SZ are significantly more resistant to the illusion than all other groups. However, no significant differences were found between the remaining groups.
Ponzo illusion	Kantrowitz et al (2009)	38 Chronic SCH/SCH-A (*M* = 37.3 years) 28 HC (*M* = 36.5 years)	N/A	40.9	Forced choice	Magnitude of the illusion weaker in patients with schizophrenia compared to controls
Poggendorff illusion	Kantrowitz et al., 2009	38 Chronic SCH/SCH-A (*M* = 37.3 years) 28 HC (*M* = 36.5 years)	N/A	40.9	Forced choice	No significant difference between patients with schizophrenia and controls
Oppel Kundt illusion	Letourneau, 1974	5 Paranoid SCH (*M* = 47.6 years) 5 Simple SCH (*M* = 46.3 years)			Size-judgment task	Patients with simple schizophrenia were significantly less affected by the illusion than paranoid SZ
Sander parallelogram	Kantrowitz et al., 2009	38 Chronic SCH/SCH-A (*M* = 37.3 years) 28 HC (*M* = 36.5 years)	N/A	40.9	Forced choice	No significant difference between patients with schizophrenia and controls
Hollow-mask illusion	Dima et al., 2009	13 SCH (2 F, 11 M, *M* = 33 years) 16 HC (3 F, 13 M, *M* = 32 years)	18.5, 19.7, 78.9	N/A	Forced choice	Magnitude of the illusion weaker in patients with schizophrenia compared to controls
	Dima, Dillo, Bonnemann, Emrich, & Dietrich, 2011	20 SCH (4 F, 16 M, *M* = 33.5 years) 20 HC (4 F, 16 M, *M* = 33.3 years)	20.2, 18.6, 80.0	N/A	Forced Choice	Patients were much less susceptible to the illusion that controls
	Emrich, Leweke, & Schneider, 1997	13 SCH (8 F, 5 M, 26–42 years) 20 HC (13 F, 7 M, 20–48 years)	N/A	39	Scored qualitative description of perception	Significantly higher index of inversion (for both familiar and unfamiliar objects) for patient group, thus showing greater illusory resistance
	Keane, Silverstein, Wang, & Papathomas, 2013	30 SCH (10 acute partial hospital patients, 10 extended partial hospital patients and 10 outpatients 9 F, 21 M, *M* = 46.6 years) 25 HC (13 F, 12 M, *M* = 45.8 years)	14.7, 17.3, 29.8	N/A	Forced choice	Magnitude of the illusion weaker in patients with simple schizophrenia compared to HC Illusory resistance was correlated with positive symptoms as measured by Positive and Negative Syndrome Scale (PANSS)
	Schneider et al., 2002	20 inpatient SCH (12 F, 8 M, M = 38.5 years) 15 HC (8 F, 7 M, M = 31.5 years)	24, 23, 93 (at 1st session)	N/A	Rating scale	Depth inversion significantly higher (i.e. greater resistance to illusion) in SCH compared to controls within a week of admission and within 3rd week. However, 1 week before discharge no apparent differences compared to controls.
Illusory line motion	Crawford et al., 2010	19 Chronic SCH (4 F, 15 M, 24–48 years) 26 HC (9 F, 17 M, 21–56 years)	N/A	N/A	Forced choice	Magnitude of the illusion weaker in patients with schizophrenia compared to controls
	Sanders, de Millas, Heinz, Kathmann, & Sterzer, 2013	34 Paranoid SCH (13 F, 21 M, *M* = 35 years) 34 HC (13 F, 21 M, *M* = 34.6 years)	18.1, 21.5, 75.3	N/A	Forced choice	Magnitude of the illusion weaker in patients with paranoid schizophrenia compared to controls Apparent motion of stimuli was negatively correlated with Peter’s Delusional Ideation (PDI) conviction subscale
Roelof effect	Chen, McBain, Norton, & Ongur, 2011	33 SCH (15 F, 18 M, *M* = 20.5 years, 17 SCH, 16 SCH-A) 34 HC (17 F, 17 M, M = 15.1 years)	15.4, 14.4, (total not reported)	N/A	Forced response	Greater illusionary effects seen in responses of patients with schizophrenia compared to healthy controls
Kanizsa square	Spencer & Ghorashi, 2014	17 SCH (1 F, 16 M, *M* = 43.8) 14 HC (2 F, 12 M, *M* = 43.6)	N/A	N/A	Forced choice	No difference in errors of whether an illusory square was present or not.
	Keane et al., 2014	75 Patients (46 SCH, 29 SCH-A, 62 % M, *M* = 46.0 years) 18 HC (50 % M, *M* = 41.1 years)	20.1, 18.2, 77.9	N/A	Forced choice	Patients were less able to distinguish between illusory and non-illusory conditions however, distractor contours reduced illusion perception similarly to as seen in HC suggesting shape perception not illusory contour difficulties.
Hermann grid	Kantrowitz et al., 2009	38 Chronic SCH/SCH-A (*M* = 37.3 years) 28 HC (*M* = 36.5 years)	N/A	40.9	Forced choice	At 100 and 50 % levels of contrast, patients were significantly less susceptible to the illusion than controls, but not at 10 or 30 %.
Contrast–contrast illusion	Barch et al., 2012	153 SCH/SCH-A (62 % M; *M* = 39.6 years) 137 HC (55 % M; *M* = 36.7 years)	N/A	Positive symptoms = 8.7, Negative symptoms = 7.4, Disorganised symptoms = 5.2	Forced choice	Reduction in the contrast-contrast effect in SCH compared to healthy controls.
	Dakin, Carlin, & Hemsley, 2005	15 Chronic SCH 20 HC	N/A	N/A	Forced choice	Patients were less vulnerable to contrast-contrast effect than healthy controls.
	Yang et al. 2013a	30 SCH (11 M, 19 F, *M* = 41 years) 23 HC (11 M, 12 F, *M* = 39 years)	N/A	13	Contrast-judgment task	Weakened effect of contrast-contrast effect in SCH compared to HC.
	Tibber et al., 2013	24 SCH (8 F, 16 M, *M* = 40.0) 24 HC (8 F, 16 M, *M* = 38.2)	14.4, 17.0, 60.9	N/A	Forced choice	Less susceptible to the illusion than HC and therefore weaker surround suppression of contextual contrast
Brightness illusion	Yang et al., 2013a	30 SCH (11 M, 19 F, *M* = 41 years) 23 HC (11 M, 12 F, *M* = 39 years)	N/A	13	Luminance-judgment task	No difference in effect of illusion on SCH in comparison to HC.
	Tibber et al., 2013	24 SCH (8 F, 16 M, *M* = 40.0) 24 HC (8 F, 16 M, *M* = 38.2)	14.4, 17.0, 60.9	N/A	Forced choice	No difference between HC and SCH

*PANSS* Positive and negative symptom scales (Kay, Fiszbein, & Opler, 1987), scores are reported for patient group (to 1 dp; +ve, -ve, total); *BRPS* Brief psychiatric rating acale (Overall & Gorham, 1962), scores are reported for patient group; *HC* healthy controls; *SCH *patients with schizophrenia; *SZ-A* schizoaffective patients

### Low-level integration illusions and schizophrenia

It has been reported that individuals with schizophrenia are resistant to some low-level integration illusions, including the contrast–contrast, luminance, Hermann grid, and line motion illusions (Table 2).

In contrast–contrast illusions, a circular patch of a fixed contrast is surrounded by a circle of a lower or higher contrast. Higher contrast surrounds result in the inner patch being perceived as having a lower contrast than its true contrast and vice versa. Individuals with schizophrenia are less likely to experience this ‘contrast–contrast’ effect and report perceiving contrasts closer to the actual contrast of the inner patch (Barch et al., 2012; Dakin et al., 2005; Tibber et al., 2013; Yang et al., 2013a). However, it is important to note that, whilst Barch and colleagues (2012) reported a reduced contrast–contrast effect, their effect size was notably smaller than Dakin and colleagues’ (2005) original study. Moreover, their effect became non-significant when lapses in attention were controlled for (Barch et al., 2012).

These findings suggest that there is some level of abnormality in the low-level integration mechanisms that combine and integrate inhibitory and excitatory responses of local neurones to distinguish boundaries between the two different contrasts. In healthy participants, these gain control mechanisms enhance the perceived contrast of one patch and weaken the other to create greater distinction between the two in order to adapt and optimise visual discrimination. In individuals with schizophrenia, there is a greater accuracy to the actual contrast and therefore the low-level integration mechanism as mediated by gain control is either weakened or not operating.

This abnormality is further demonstrated in the Hermann grid illusion where gain control causes dark patches to appear along the white lines of the grid, inbetween the darker squares. Those with schizophrenia also show a resistance to this illusion compared to controls, but only at higher levels of contrast (50 and 100 %) but not at lower (10 or 30 %) where no significant differences were found (Kantrowitz et al., 2009). Another low-level integration illusion where there is an apparent resistance is illusory line motion. Both studies identified by this review found that patients were significantly less susceptible to the illusion than healthy controls (Crawford et al., 2010; Sanders et al., 2013). Specifically, Sanders and colleagues (2013) found the magnitude of this illusion to be inversely correlated with Peter’s Delusional Ideation conviction subscale. In other words, resistance to the illusion increases as a function of strength of an individual’s belief that their delusions are veridical. This not only suggests that the visual mechanisms underlying this illusion are in deficit but also suggests that this abnormality is linked to the disorder. These abnormalities in low-level integration could have a major impact on how people with schizophrenia perceive the basic features of visual scenes, such as edges and contrasts, potentially providing them with a fundamentally altered perception of the world compared to the general population (Kantrowitz et al., 2009). Therefore, they may, to some degree, explain some level of anomalous perceptual experience in schizophrenia.

Interestingly, this early disruption in visual processing has not been observed in other types of low-level integration illusions. Studies examining brightness illusions have shown no differences in susceptibility between people with schizophrenia and healthy controls (Tibber et al., 2013; Yang et al., 2013b). If one considers that susceptibility to brightness, but not contrast illusions, is the same between these two population groups, then one can infer that the neural mechanisms responsible for brightness perception remains unaffected in schizophrenia whilst those neurons that code contrast are in fact implicated by the illness, suggesting against a generalised impairment in low-level integration. One possibility is that the local circuitry mediating gain control for detecting contrast boundaries is not affected in schizophrenia per se but rather the mechanisms of top–down modulation on this circuitry.

Tibber and colleagues (2013) proposed that preserved judgments of luminance, but not contrast, indicate that luminance is processed pre-cortically, before the signals reach V1. Therefore, any deficits to low-level integration would be constrained to V1, rather than to earlier structures. However, this claim is contradicted by the findings of the Hermann grid illusion outlined previously, where at lower contrasts there was no apparent resistance to the illusion (Kantrowitz et al., 2009).

Some low-level integration mechanisms seem to be abnormal in schizophrenia. However, the dissociation between the contrast and brightness illusions despite their dependence on similar gain control mechanisms suggests that specific abnormalities are present rather than deficits to general low-level processes.

Further research is needed to more specifically identify which types of low-level integration mechanisms are affected in schizophrenia and whether these are cortically or pre-cortically mediated. Moreover, future research should investigate the effects of other classical low-level integration illusions, such as illusory Mach bands, whereby the illusion produces a similar contrast–contrast effect as the contrast illusions using strips of colour rather than surrounds. This could provide confirmatory results to support the findings for other contrast–contrast illusions using different mechanisms.

### High-level integration illusions and schizophrenia

Many studies have been conducted to corroborate the existence of abnormal perceptual organisation by weak contextual integration in schizophrenia. One way in which this has been achieved is using visual illusions that are more cognitively demanding for understanding their context. The Ebbinghaus, Ponzo, Müller-Lyer and Poggendorff illusions conceivably fall into this category. For example, a popular account for the Ebbinghaus illusion (Table 1) entails a size contrast effect whereby the relative size of the surrounding circles is mentally compared to the size of the inner circle, making the latter appear smaller when the former is larger, and vice versa (Coren & Enns, 1993). It then follows that problems in processing the contextual elements of the visual scene will diminish the magnitude of the Ebbinghaus illusion in schizophrenia. This seems to be the case. Uhlhaas and colleagues (2006b) demonstrated that individuals with greater disorganised schizophrenia symptoms are less susceptible to the Ebbinghaus illusion than individuals with non-disorganised symptoms. A similar finding was also reported by Tibber and colleagues (2013). However, Yang et al. (2013a) did not replicate this finding and reported no significant differences between those with schizophrenia and healthy controls.

There appears to be more consistency with the hollow-mask illusion. Dima and colleagues (Dima et al., 2009; Dima, Dietrich, Dillo, & Emrich, 2010; Dima et al., 2011), Keane et al. (2013) and Emrich, Leweke and Schneider (1997) have shown that people with schizophrenia are considerably less susceptible to the hollow-mask illusion compared to control participants. However, Schneider and collaborators (2002) reported that the resistance compared to healthy controls was found at 1 and 3 weeks after admission for treatment, but was not apparent in their patient sample 1 week before discharge. This, coupled with lower mean PANSS (positive and negative symptom scales) scores at this testing point, suggests that such resistance is state-specific.

PANSS scores may explain some of contradictory findings in the literature. For example, Yang and colleagues (2013a) did not replicate the results observed in other studies using the Ebbinghaus illusion. They described their participants as clinically stable at the time of testing. In contrast, Ulhass and colleagues (2006b) showed a greater resistance to the same illusion in those with greater disorganised symptoms, as outlined above. Those with disorganised symptoms also had significantly greater positive, negative and total psychopathology as measured by the PANSS than the non-disorganised group. This suggests an association between abnormal task performance and a lack of cognitive coordination experienced as part of the disorganisation syndrome (Uhlhaas et al., 2006b). However, when the schizophrenia groups were pooled, correlations were found with both the cognitive and disorganised PANSS factors. This lends support to the hypothesis that resistance to the Ebbinghaus illusion is only present in more acutely ill samples, rather than the more stable group tested in the Yang et al. (2013a) study. However, there is evidence of these abnormalities being a longitudinal factor in the disease process of schizophrenia, with effects being found in both chronic samples (e.g. Dakin et al., 2005; Kantrowitz et al., 2009) and non-clinical schizoptypal samples (Bressan & Kramer, 2013; Uhlhaas, Silverstein, Phillips, & Lovell, 2004). Further experimentation is required to tease apart the state-specific resistance outlined here.

Although most studies report similar results, some findings do not support the hypothesis that patients with schizophrenia are less susceptible to this class of visual illusions. The schizophrenia literature is quite inconsistent with respect to the susceptibility to the Müller-Lyer illusion, a frequently studied visual binding task (Table 2). Whilst a number of studies have found no effect of the illusion in patients with schizophrenia (Parnas et al., 2001; Tam et al., 1998), others have provided evidence of increased susceptibility (Kantrowitz et al., 2009; Parnas et al., 2001; Weckowicz & Witney, 1960) and none provided evidence of decreased susceptibility, as far as we know. These discrepancies in findings could be due to a number of issues with previous work in this field that are discussed in section 3.

In the case of the Kanizsa square illusion, there is also no definite resistance in those individuals with schizophrenia. Keane and colleagues (2014) found that patients were less able to distinguish non-illusory and illusory stimuli in the Kanizsa square illusion than controls (see Table 1 for description), suggesting that they were less susceptible to the illusory effects and therefore unable to discriminate between the two conditions. Nonetheless, when disruptor contours were introduced to interfere with illusory discrimination, there were no differences between patients and the appropriately matched control groups. This suggests that the deficit was related to a higher-level shape perception process rather than a lower-level contour formation mechanism. However, whilst Keane and colleagues (2014) found an effect of schizophrenia on the perception of illusory squares, their result was not significantly replicated in a different study (Spencer & Ghorashi, 2014).

For some illusions, it is hard to assess the nature of how they are perceived by those with schizophrenia, simply due to the seeming lack of testing of these types of illusions. For example, there is only a single study which assesses the Ponzo, Poggendorf and Sander parallelogram illusions (Kantrowitz et al., 2009). In this case, only the Ponzo illusion seemed to be weakened by schizophrenia. However, this may not be evidence against enhanced resistance to visual illusions in schizophrenia. These findings are limited due to their lack of replication. Similar difficulties arise with the Oppel-Kundt illusion where the only study of the illusion on individuals with schizophrenia provided no healthy control group with which to compare findings (Letourneau, 1974) .

To summarise, the evidence reviewed suggests a general trend of an increased resistance to high-level visual illusions. However, it will be important to solidify these findings through the replication of results, specifically across a single sample, in order to provide a clearer picture of the illusions to which those with schizophrenia may be resistant.

### Visual illusions and perceptual organisation in schizophrenia

Dima and colleagues (2011) proposed that visual illusions can be divided into two categories: those which are physiologically based and those which are cognitively based. Their division is similar to the low-level versus high-level dichotomy presented in this review.

Whilst people with schizophrenia typically show decreased performance in cognitive tasks, it is interesting to note a more accurate performance in perceptual judgment tasks using visual illusions compared to controls. We can therefore assume the existence of a specific processing abnormality in these patients rather than an artefact of a generalised cognitive deficit (Dakin et al., 2005) or of low-level visual problems, given that some but not all illusions are affected. The hypothesis of a specific processing abnormality in schizophrenia has been supported by a number of visual illusion studies that have matched participants using general IQ measures and still found significant differences in illusion perception for people with schizophrenia (Keane et al., 2014; Tibber et al., 2013) .

Overall, these findings looked at visual illusions which modulate the perceptual experience of individuals using both high- and low-level integration mechanisms. However, there seems to be a trend in the literature suggesting that people with schizophrenia are more resistant to higher-level integration illusions which require contextual and more complex cognitive operations in order to produce their illusory effects. This suggests a disruption to the top–down perceptual organisation mechanisms that operate to integrate higher-level information, such as context, in order to modulate the initial lower-level percept.

Importantly, neurophysiological evidence has supported the role of reduced top–down modulation in schizophrenia, specifically with regards to visual illusions. Dima and colleagues (2009, 2010) have found, using dynamic causal modelling (DCM) with both ERP and fMRI data, that individuals with schizophrenia exhibit different modulations of neural connectivity during the presentation of the hollow-mask illusion compared to healthy controls. In particular, data from schizophrenia samples showed a preference for a DCM model of forwards connections from V1 to the lateral occipital cortex (LOC). This suggests a bottom–up processing approach which ‘ignores’ top–down influences, such as contextual cues from higher-level brain areas. In contrast, healthy controls exhibited task modulation in the backwards connection from the intraparietal sulcus (IPS) to the LOC. Because IPS and LOC are in two completely different cortical streams of visual processing, with IPS being in the dorsal stream and LOC being in the ventral stream, this finding reflects some kind of top–down control over the visual system. When coupled with behavioural data for the hollow-mask illusion (Dima et al., 2009, 2010, 2011; Keane et al., 2013), it can be argued that top–down control mediates the perceptual experience of visual illusions in healthy controls, whereas people with schizophrenia are resistant to these illusory effects due to a reduction in top–down control.

## Limitations in previous research

Discrepancies in results may be related to methodological issues, such as the heterogeneity of the sample (Pessoa, Monge-Fuentes, Simon, Suganuma, & Tavares, 2008). Therefore, when carrying out visual psychophysics studies in patients with schizophrenia, future research should take into account a number of potential confounding factors, including medication effects, task response types, the number of illusions tested and disease heterogeneity.

### Medication as a confounding variable

Until the 1990s, research on cognitive deficits in schizophrenia had typically been conducted with chronically ill patient groups (Mesholam-Gately, Giuliano, Goff, Faraone, & Seidman, 2009), with the majority of samples having significant medication and treatment exposure (Heinrichs & Zakzanis, 1998). Thus, the role of medication effects and illness course on visual processing should be considered. Conducting visual illusion research with individuals at an earlier stage of illness, or with those at risk of developing psychosis, may be useful in providing further insights, as this will avoid the potential confounding nature of these types of variables.

Medication effects, such as use of antipsychotic or mood-stabilising medications, must be taken into account when investigating cognitive functions in schizophrenia (Stip, 2006). More specifically, links have been found between medication dosages and performance in visual illusions. Diržius et al (2013) observed significant differences in the strength of the Müller-Lyer illusion between a paranoid schizophrenia group and controls for only 5 out of 40 presentations of the illusion. Significant differences in illusion strength were also found between chlorpromazine-equivalent dosages when the testing sessions were separated based upon medications taken that day. The direction of these effects was not recorded in this published pilot study. However, the group with the highest chlorpromazine dose equivalents of antipsychotics showed the greatest perceptual errors, suggesting they were more susceptible to the illusion. Therefore, medication as a confounding variable could explain contradicting results such as those previously discussed regarding susceptibility to the Müller-Lyer illusion, but further testing is required to predict in what direction or magnitude it may affect the results.

### Forced choice response task

Another issue in previous methodologies is the frequent use of a forced choice response to measure susceptibility to visual illusions. These types of tasks involve having the participant indicate which of two target stimuli is larger or having the participant indicate if two stimuli are the same or different. The majority of the findings reported in Table 2 arise from a forced choice design. However, it has been argued before that this kind of design is suboptimal for the purposes of measuring perception. A forced choice response involves mentally recreating subjective threshold criteria of size difference which the illusion must break before an illusion-supporting response is reported (Skottun & Skoyles, 2014). When making between-group comparisons, this may affect the validity of inferences made about the visual systems of those groups; such as patients with schizophrenia. Therefore, alternative measurements of perceptual judgment such as manual estimations, adjustments and match-to-sample tasks, could be used instead to try and reduce the confounding nature that previous forced-response tasks may have introduced in visual illusion research. However, a more general critique of the use of visual illusions in studying perception has been offered in the literature (Skottun & Skoyles, 2014): as illusions are necessarily based on subjective criteria, when comparing performance in two groups (i.e. patent vs. control group), one cannot be certain whether differences in susceptibility reflect differences in the response criteria or differences in the underlying sensory process, making it difficult, if not impossible, to apply criterion-independent tests.

### Studying illusions in isolation

Many of the studies presented in this article investigated either a single or a small number (all *n* < 3 except Kantrowitz and colleagues 2009) of visual illusions; (e.g. Dima et al., 2009; Sanders et al., 2013; Uhlhaas et al., 2006b). Whilst it is important to draw conclusions on the effects of schizophrenia on illusion perception from the research literature as a whole, this can be difficult for several reasons. Due to the small number of illusions tested in each study, comparisons of the effects of schizophrenia on low-level and high-level integration illusions have to be made across multiple studies to achieve a complete picture of these effects. This can call into question between group comparisons, especially due to the issues the heterogeneity of these samples introduces (Pessoa et al., 2008).

### Heterogeneity of disease

As psychotic symptoms occur within several Diagnostic and Statistical Manual of Mental Disorders (American Psychiatric Association, 2013) diagnostic categories, a single-symptom (Persons, 1986) or dimensional approach may be useful in studying perceptual anomalies in schizophrenia and related psychotic disorders. Studying visual illusion perception across diagnostic categories of psychotic disorders or symptom dimensions may link specific psychotic symptoms (e.g. hallucinations) with underlying neurobiological mechanisms, and help deconstruct the heterogeneity of disorders like schizophrenia. Anomalies of perception may also be an important feature for at-risk mental states and prodromal psychosis (Yung & McGorry, 1996). Furthermore, visual perceptual disturbances, such as symptoms of visual distortion and hallucinations, are frequently found in neurological disorders such as Parkinson’s disease (Archibald, Clarke, Mosimann, & Burn, 2011). If specific links were to be made between performance on visual illusion tasks and specific psychotic symptoms (e.g. visual hallucinations or distortions), it would therefore be useful to compare performance on illusion paradigms in individuals with and without hallucinations, or to repeat paradigms in individuals when they are experiencing acute hallucinations and when such symptoms have remitted. Whilst the current paper focuses on visual perception in schizophrenia, it is possible that by adopting a dimensional approach, similar abnormalities in perceptual processing may also underpin neurobiological processes occurring outside of schizophrenia.

Another disorder which has been linked to visual illusion research is autism. Both schizophrenia and autism share similar symptomologies of social impairment and anomalous perceptual experience (American Psychiatric Association, 2013), and even similar genetic overlap (Burbach & van der Zwaag, 2009; Carroll & Owen, 2009). Recent research has outlined a possible overlap between schizotypal and autistic tendencies, particularly for a disorganised phenotype, using factor analysis (Ford & Crewther, 2014). Disorganised symptoms of schizophrenia have been highly correlated with susceptibility to illusions (Horton & Silverstein, 2011; Uhlhaas et al., 2006b), whilst research in autism has produced mixed results with some studies demonstrating a resistance to visual illusions (Bölte, Holtmann, Poustka, Scheurich, & Schmidt, 2007; Happé, 1996) but not others (Hoy, Hatton, & Hare, 2004; Ropar & Mitchell, 1999, 2001). It is therefore possible that atypical perceptual experiences may be mediated by these types of disorganised symptomologies. However, Ford and Crewther (2014) found that unusual perceptual experience contributed more highly to a perceptual oddities factor rather than the disorganised factor, suggesting that the relationship between these two diagnoses and susceptibility to illusions is not simple, and probably encompasses a number of interacting variables. In order to ascertain that the correlations between schizophrenic symptomologies and resistance to visual illusions are mediated by a common neural mechanism, cross-diagnostic research may be useful.

## Bridging abnormal perception and psychotic symptoms

The reduced susceptibility to visual illusions in people with schizophrenia has been correlated with a number of psychotic symptoms, such as disorganisation (Horton & Silverstein, 2011). For example, in a community sample measured on their level of schizotypy, thought disorder was found to be associated with visual illusion susceptibility (Uhlhaas et al., 2004). This again may highlight the role of perceptual organisation in the experience of visual illusions, particularly considering the significant relationships between cognitive disorganisation symptoms and perceptual organisation that have been found (Feigenson, Gara, Roché, & Silverstein, 2014). Therefore, it may be the case that the general impairment to perceptual organisation in schizophrenia (Silverstein & Keane, 2011a) mediates these correlations with the disorganisation syndrome. Further research will be needed to investigate this. However, we explain in this section how we think examining the reduced susceptibility to visual illusions may also shed light on the underpinnings of other psychotic symptoms, specifically visual distortions.

Hallucinations are one of the defining features of schizophrenia. Anomalies of perception are also an important feature of at-risk mental states and prodromal psychosis (Yung & McGorry, 1996). Some have attempted to reconcile the findings from studies on visual illusion with hallucination symptoms (Notredame et al., 2014). As discussed, contextual processing is an important factor for driving susceptibility to higher-level illusions such as the Ponzo illusion. Aberrations in this mechanism, as a number of researchers have argued (Glazer, Mason, King, & Brewin, 2013; Steel, Fowler, & Holmes, 2005), can also yield to visual hallucinations..

Another possible explanation to bridge symptoms and visual illusions is a Bayesian prediction approach (see Notredame, Pins, Deneve, & Jardri, 2014 for further discussion on this topic). However, one issue with this thinking is that, in order to make Bayesian predictions, the brain must first be fed sensory input. Yet, hallucinations are generated internally (Chouinard & Miller, 1999) and require no formal stimulus to be present in relation to their perceptual experience. In other words, there seems to be an absence of sensory input to produce this aberrant perception (Waters et al., 2014).

We contend that the two perceptual phenomena are distinct. Although both phenomena represent departures from reality, visual illusions are the results of erroneous interpretations by the brain of the incoming input, while hallucinations are typically referred to as 'false perception' as they are experienced regardless of external stimuli. With these opposing features in mind, an interesting question is whether or not the two forms of perceptual experiences could be linked and driven by similar mechanisms, particularly considering the abnormal nature of both these phenomena.

An alternative link to be investigated is symptoms of visual distortion experienced by patients with schizophrenia. Much like visual illusions, visual distortions are stimulus-driven with the perceptual experience of the individual directly linked to actual visual input. In other words, the resultant perceptual aberrations are linked to the inherent properties of a stimulus, for example a hat stand which is straight being perceived as more curved or bowed in shape. Interestingly, visual distortions have also been described in neurological disorders, such as migraine (Vincent, 2015) and Parkinson's disease (Archibald et al., 2011).

There may be similar neural mechanisms underlying both visual distortion symptoms and reduced susceptibility to visual illusions such as presented in this paper. This is because the fundamental premise of both is that they require misperceptions of the visual input of the outside world, with the focus here being that the visual input is required. However, further enquiry is hard to conceptualise due to a lack of literature regarding visual distortions in schizophrenia. Even when only considering the literature presented here, there is a clear trend that those individuals with schizophrenia can perceive external stimuli differently to healthy controls. Therefore, further research would be worthwhile to understand the relationship between visual distortions as symptoms experienced by patients with schizophrenia and visual illusion misperceptions induced by the methodology presented in this paper.

Hemsley’s cognitive model of psychosis (Hemsley, 2005) supports the idea of weakened top–down perceptual organisation. According to Hemsley, individuals with psychosis have a more generalised deficit in their ability to use context. In particular, it has been suggested that the sensory input does not activate stored material in people experiencing psychosis. As a consequence, attention is focused on the wealth of individual details in the environment, rather than the “bigger picture”. As past experience may help us to differentiate relevant from irrelevant stimuli, a reduced ability to access this information in people with psychosis could result in information usually outside of consciousness intruding into awareness, resulting in a feeling of salience. Clinically, this means that individuals with psychosis may have a different perceptual experience of the world, with an increased likelihood of irrelevant stimuli intruding into awareness and feeling personally relevant or important (Hemsley, 2005) This is echoed in the findings with visual illusions, with the apparent resistance to these displays reflecting a maladaptive tendency to ignore the context, which results in the illusory properties of the display.

Visual perceptual impairments, such as the maladaptive perception revealed by visual illusions, may also have a widespread effect on higher functional outcomes of schizophrenia, such as social cognition and community functioning (Green, Horan, Mathis, & Wynn, 2013). For example, significant positive correlations between biological motion perception anomalies and social perceptual ability have been found in patients with schizophrenia (Brittain, Ffytche, McKendrick, & Surguladze, 2010). These links with functional outcome may be the result of impairments in underlying perceptual mechanisms that affect subsequent higher cognitive processing (Javitt, 2009). In other words, social interactions are mediated and dependent on visually sensing dynamics such as facial features and emotions or action-based goals and intentions. Hence, it may be appropriate to assume that visual perceptual anomalies could lead to inappropriate reactions to social interactions, impairing higher-level functional outcomes and causing the widespread disruption to day-to-day functioning that those with schizophrenia may experience.

## Closing remarks

This review highlights the potential utility of visual illusions as a method of understanding the abnormal mechanistic properties of the visual system in schizophrenia. Individuals with schizophrenia seem to show a trend towards resistance to high-level integration illusions, whilst showing specific resistance to only some low-level illusions. Together, these observations seem to highlight problems in communicating between different levels of processing for certain forms of cognitive/perceptual operations. The necessary decomposition of the visual abnormalities of schizophrenia, especially between low- and high-level integration deficits, is critical to the understanding of the psychological abnormalities in perceptual organisation, which may underlie atypical perception in this illness.

However, visual perception is a highly complex process that requires a number of interlinking processes, including both low-level and higher-level integration mechanisms. Whilst studying these processes in isolation provides us with a deeper understanding of the underlying neural abnormalities in schizophrenia, it will become increasingly more important to investigate the integrative processes by which multiple top–down or bottom–up processes converge in visual perceptual organisation, and the degree to which they contribute to the overall percept. It may be some time before there are paradigms to quantifiably test these types of links. As research has shown, experience contributes largely to our perceptual experiences, either through modulation by affect (Colzato, van Wouwe, & Hommel, 2007) or through environmental input during key developmental periods (Fox & Wong, 2005; Le Grand, 2001). Therefore, it is important that we do not isolate vision research in schizophrenia from experiential modulation, through ecologically valid stimuli, as an appreciation of the system as a whole, rather than of its individual parts (Findlay & Thagard, 2012).
